# Delivery of Catechins from Green Tea Waste in Single- and Double-Layer Liposomes via Their Incorporation into a Functional Green Kiwifruit Juice

**DOI:** 10.3390/molecules28020575

**Published:** 2023-01-06

**Authors:** Weerawich Athirojthanakij, Ali Rashidinejad

**Affiliations:** 1 School of Food and Advanced Technology, Massey University, Private Bag 11222, Palmerston North 4442, New Zealand; 2 Riddet Institute, Massey University, Private Bag 11222, Palmerston North 4442, New Zealand

**Keywords:** liposomal encapsulation, catechins, green tea waste, green tea waste extract, green tea waste catechins, functional kiwifruit juice

## Abstract

Globally, about one million tonnes of tea products, which contain high concentrations of catechins and their derivatives, are wasted annually. Therefore, green tea waste catechins (GTWCs) are worth extracting, processing, protection, and delivery to the human body. In this study, GTWCs were extracted using a green method and then encapsulated in both single- (SLLs) and double-layer liposomes (DLLs). The encapsulated extracts were subsequently incorporated into a fresh green kiwifruit juice. SLLs and DLLs containing GTWCs had a size of about 180 and 430 nm with a zeta potential of −35 and +25 mV, respectively. Electron microscopy illustrated the separation of the SLLs and fibre in kiwifruit juice and attraction of the DLLs to this fibre. Liposomal GTWCs were effectively maintained in the kiwifruit juice during the 28 days of storage (4 °C), demonstrating the effectiveness of this delivery system for high-value bioactives (i.e., catechins) from such a by-product (i.e., green tea waste).

## 1. Introduction

Tea (*Camellia sinensis*) was accidentally discovered around 6000 years ago in Ancient China and was considered as a medicine to cure a variety of illnesses such as headaches, fever, and indigestion [1,2]. Green tea is a product that is obtained from the tea leaves of *Camellia sinensis* without fermentation [1,3]. Although there have been numerous tea products developed for consumption to date, about one-third of tea (one million tonnes) is wasted during harvest and processing [4,5]. To minimise such a huge waste, it is usually transformed into a feed additive, an activated carbon, a fertiliser, or a low-cost absorbent, which do not gain the maximum benefit of such a high-value product. One of the most efficient ways for the valorisation of tea waste is to extract its bioactive compounds, which could be used as food additives and/or supplements in health-promoting products such as functional foods or nutraceuticals [6].

Polyphenols in green tea mainly consist of phenolic acids and catechins, especially (−)-epigallocatechin-3-gallate (EGCG), which provides the highest potential of antioxidant activity when compared to other catechin derivatives including (+)-catechin (C), (−)-epicatechin (EC), (−)-epicatechin-3-gallate (ECG), and (−)-epigallocatechin (EGC) [7]. These polyphenols play an important role in providing the human body with an increased level of antioxidants, decreasing oxidative stress, and subsequently, reducing the risk of chronic diseases such as cancer, diabetes, cardiovascular, neurodegenerative, and Alzheimer’s disease [8]. Moreover, several clinical studies have proven that EGCG can prohibit the occurrence and multiplication of tumour in various sites such as the stomach, skin, liver, lung, and colon. Gupta et al. [9] reported that EGCG had the potential to induce apoptosis and promote cell growth, which in turn can lead to the alteration of the protein cell cycle expression, the deactivation of nuclear factor kappa B (NFκB), and the activation of caspases. In addition, EGCG affects the transcription of tumour suppressor genes because it can reduce the activity of DNA methyltransferase, dihydrofolate reductase, and proteases [10]. However, it is difficult to maintain the functionality/bioactivity of green tea catechins in food products due to the instability of catechins under both environmental (during processing and storage) and gastrointestinal tract (GIT) conditions. Therefore, an effective delivery system needs to be applied for the protection of catechins against such conditions and their controlled release in the GIT [3]. 

Encapsulation technology has been developed as a delivery approach to retain valuable bioactive substances (known as core materials) by their entrapment in the coating materials (also known as shell, carrier, or encapsulant) [11]. There are several encapsulation methods such as freeze drying, spray drying, emulsions, nanoprecipitation, and liposomes that are suitable for different kinds of bioactive materials. In terms of catechin encapsulation, liposomes are known as a suitable and efficient method, because they are flexible carriers with amphiphilic, biocompatibility, biodegradability, and non-toxicity properties [3,12]. Various studies have reported the manufacture of single-layer liposomes (SLLs) containing green tea catechins. Rashidinejad, Birch, Sun-Waterhouse, and Everett [3] demonstrated the delivery of green tea catechins, which were encapsulated in SLLs from soy lecithin and incorporated into a low-fat hard cheese. The SLLs could protect the bioactives, leading to the increase in antioxidant activity and phenolic content in the corresponding cheese samples. Chen et al. [13] manufactured SLLs to co-encapsulate EGCG and quercetin for an increment in their antioxidant activity due to a synergistic effect between two bioactives. However, systematic studies about the entrapment of green tea catechins in double-layer liposomes (DLLs) are scarce and none have been published on the use of these vehicles for the delivery of catechins from green tea waste. Therefore, this study aimed to reveal the effectiveness of DLLs in comparison to SLLs.

Kiwifruit, which belongs to *Actinidia* genus with many cultivars, has been used for human consumptions for a long time. There exist two major cultivars for this fruit including *A. deliciosa* and *A. chinensis*, which can be easily identified by the “green” and “gold” colour of fruit pulp, respectively [14]. In terms of the compositions, the fruit contains water (approximately 80 g/100 g raw fruit) in combination with carbohydrate, sugar, fibre, protein, fat, and vitamin C (14, 9, 3, 1, 0.5, and 161 mg/100 g of raw fruit, respectively) [15]. Although kiwifruit itself contains catechins (about 3.01 to 7.03 mg catechin equivalent/100 g fresh weight), this concentration is much lower than the human daily requirement (28 mg/100 g) [8,16]. For these reasons, kiwifruit is considered as an appropriate delivery vehicle for antioxidants such as green tea catechins, where such an approach can add functional values to the products obtained from this fruit (e.g., kiwifruit juice). Therefore, the current study aimed to study: (i) the protection and stabilisation of the extracted catechins using single- (SLLs) and double-layer liposomes (DLLs); (ii) incorporation of the manufactured liposomes containing catechins into a kiwifruit juice; and (iii) behaviour of the encapsulated catechins in kiwifruit juice (both raw and pasteurised) and their effect on various properties of this juice.

## 2. Results

### 2.1. The Behaviour of the Single-Layer Liposomes (SLLs) in Green Kiwifruit Juice and the Effect on Its Properties

#### 2.1.1. Encapsulation Efficiency (EE) and Loading Capacity (LC)

The EE in this experiment was about 67.85 ± 0.88%, which corresponded to the results of Rashidinejad, Birch, Sun-Waterhouse, and Everett [3], who reported the liposomes of green tea catechins incorporated into low-fat hard cheese. However, these results were lower than the corresponding values reported by Zou et al. [17], and higher than those reported by Ersus and Yurdagel [18]. A possible explanation for the discrepancy between these values may relate to the differences in the encapsulation technique and the type of encapsulant.

The LC represents the maximum catechins capsulated in the liposomes. In this study, the LC of the encapsulated catechins was about 37.12 ± 1.07%. This value is similar to the results reported by Ahmad et al. [19] and lower than the findings of the study carried out by Rashidinejad, Loveday, Jameson, Hindmarsh, and Singh [20]. The difference in the LC might be linked to the differences in the encapsulation method and the coating material. Moreover, the LC value is related to the EE, where it can be assumed that the greater the EE, the higher the LC.

#### 2.1.2. Particle Size and Zeta Potential

**Figure 1:**
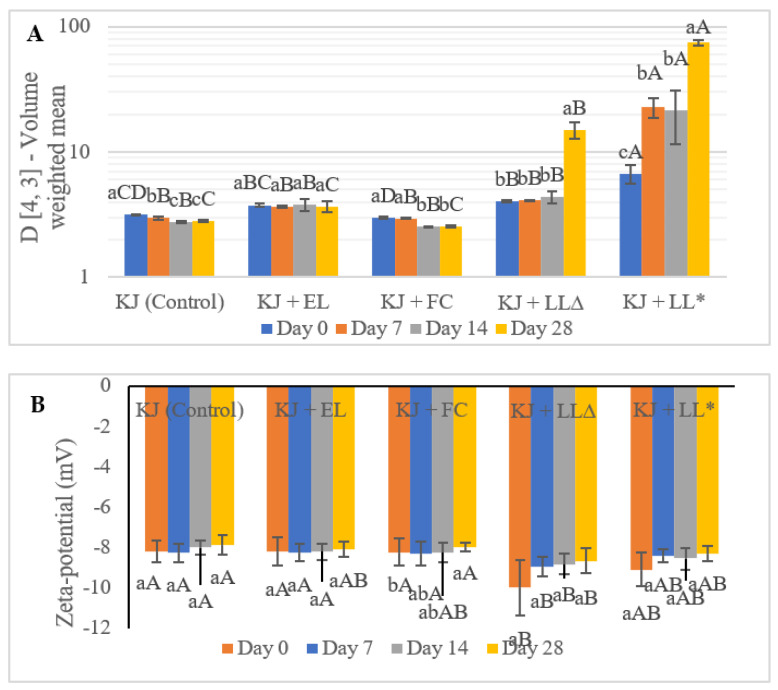
The particle size (D [4, 3]-Volume weighted mean (µm)) (**A**) and the zeta potential (**B**) of kiwifruit juice containing empty and green tea waste extract (GTWE)-loaded single layer liposomes (SLLs) during storage (4 °C). KJ: control kiwifruit juice; KJ + FC: kiwifruit juice containing free GTW catechins; KJ + LL∆: unpasteurised kiwifruit juice enriched with GTWE-loaded SLLs; KJ + LL*: pasteurised kiwifruit juice containing GTWE-loaded SLLs. Different lowercase letters indicate significant (*p* < 0.05) differences among the same kiwifruit juice samples for different storage times; Different capital letters stand for statistically significant (*p* < 0.05) differences among different kiwifruit juice samples on the same storage day.

The particle size (D [4, 3]-Volume weighted mean) of kiwifruit juice fortified with SLLs was determined to investigate the stability of the liposomes suspended in the kiwifruit juice during the 28 days of storage at 4 °C. According to the data presented in Figure 1A, the particle size of the control kiwifruit juice and kiwifruit juice containing free GTWCs significantly decreased (*p* < 0.05) after 14 days of storage. Such a reduction in the particle size of both samples may be linked to the activity of pectin methylesterase (PME), which is considered as a cause of cloud loss and gelation problems in fruit juice processing [21]. PME is a catalyst related to the hydrolysis of homogalacturonan (part of pectin polysaccharide). During the hydrolysis process, the methoxy and carboxylic groups are released and result in the production of free radicals that can decrease the particle size and molecular weight [21,22]. The kiwifruit juice containing empty liposomes had a consistent particle size until the end of storage.

In terms of the kiwifruit juice enriched with the SLLs, the pasteurisation process was carried out to investigate the stability of the liposomes under pasteurisation conditions by comparing the results of the pasteurised and unpasteurised kiwifruit juice samples. The particle size of the unpasteurised kiwifruit juice was not significantly affected (*p* > 0.05) from Day 0 to Day 14 although significantly increased (*p* < 0.05) on Day 28. However, the particle size of the pasteurised kiwifruit juice significantly shifted (*p* < 0.05) to the larger particles since Day 7, which was bigger than the size of the unpasteurised kiwifruit juice on Day 28, and then soared up to the biggest particles among all kiwifruit juice samples on Day 28 (about 74 µm). This might be related to the effect of pasteurisation, the presence of calcium ions (Ca^2+^), and the interaction between the fibre and polymer chains. Nonetheless, in this study, we only tested the effect of one pasteurisation method, indicating that the effect of other pasteurisation methods/heat treatment is unknown. Typically, raw green kiwifruit contains calcium (approximately 34 mg/100 g FW), which may be present in the kiwifruit juice after squeezing [15]. The presence of Ca^2+^ can play a crucial role in the interactions between particles that result in the aggregation of liposomes, because the dehydration of the bilayers could take place due to the induction of Ca^2+^. This reaction could decrease the number of water-binding sites on the bilayers, which in turn, could result in the decrease in repulsive hydration forces, and then the particles could easily aggregate (because of the lower repulsion force). Nevertheless, thermal treatments such as pasteurisation may enhance such a phenomenon [23].

Sun-Waterhouse et al. [24] added apple fibre and carboxymethylcellulose (CMC) (as a stabiliser) to smoothie beverages to enhance the nutritional value and prevent the breakdown of the emulsion system. The fortified beverages were then pasteurised at 85 °C for 15 s before the investigation of feasible interactions. These researchers [24] reported that an increase in the particle size of fibre could be related to the interactions between the fibre and polymer chains. The CMC polymer chains could interact with the fibre particles and then the CMC chains were loosened and coated the particles of apple fibre on the external surface, resulting in the increase in particle size and decrease in viscosity. This interaction can be the reason for the increase in the particle size of both the pasteurised and unpasteurised kiwifruit juice samples during 28 days of storage in the current study. Because some SLLs might become degraded over time and destroyed during the pasteurisation process, this may lead to the disentanglement of the phospholipids (becoming polymer chains) with the possibility to react with fibre in the kiwifruit juice.

According to Figure 1B, the zeta potential of all kiwifruit juice samples was not significantly (*p* > 0.05) different during the 28 days of storage, except for the pasteurised kiwifruit juice fortified with the SLLs. The kiwifruit juice samples with no liposomes, control kiwifruit juice, and kiwifruit juice fortified with free GTWCs were stable within a certain period of storage. This was due to the consistency of the zeta potential values (−8.28 ± 0.46 to −7.89 ± 0.49 and −8.31 ± 0.59 to −7.97 ± 0.20 mV, respectively) throughout the storage period, in combination with the smallest particle sizes reported previously. To the best of our knowledge, there is no supporting evidence in the published literature about the zeta potential of kiwifruit juice. Therefore, the findings of this experiment were the first evidence to reveal that the zeta potential of the control kiwifruit juice was about −8 mV and the addition of free GTWCs in kiwifruit juice did not change the zeta potential.

The kiwifruit juice containing empty liposomes also showed compatible zeta potential values in the range of −8.26 ± 0.44 to −8.08 ± 0.37 mV, together with the small particle size shown previously. This value was similar to the results of the kiwifruit juice samples with no liposomes, where it could be assumed that the addition of empty liposomes did not affect the zeta potential value of the kiwifruit juice. Both pasteurised and unpasteurised kiwifruit juice samples containing the SLLs presented higher zeta potential values (−9.10 ± 1.39 to −8.31 ± 0.62 and −10.00 ± 0.84 to −8.66 ± 0.36, respectively) than the other juices because of the effect of the liposomal enrichment [25]. The addition of the SLLs elevated the population of particles in the solution, which reduced the distance between particles, while it also increased the inter-particle interactions linked to the zeta potential. The lower the size of the suspended particles in the solution, the greater the zeta potential was achieved, which resulted in the colloidal stability of the system. Moreover, these results were partially in agreement with the findings of Rashidinejad, Birch, and Everett [12], who determined the zeta potential of the dispersions of milk fat globules containing different concentrations (125, 250, 500, and 1000 ppm) of catechins (C and EGCG) and green tea extract (GTE). These scientists [12] reported that the higher the concentrations of EGCG and GTE added to the milk fat, the higher the zeta potential because of the interactions between the milk fat globule membrane and green tea catechins, which resulted in the alteration of phospholipid affinity. Additionally, the increase in the particle size of both samples during storage slightly decreased the zeta potential values [12].

#### 2.1.3. Morphology

**Figure 2:**
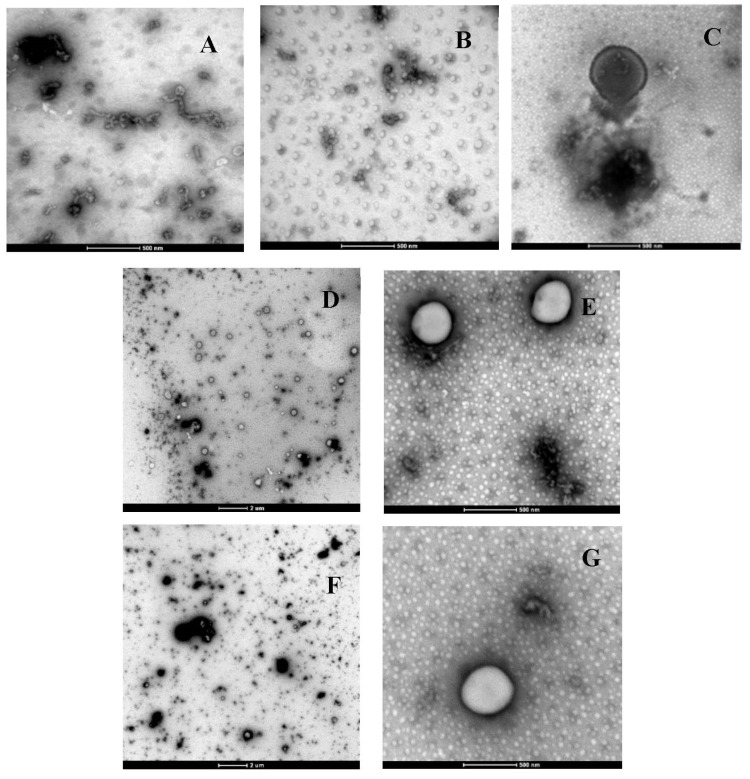
Transmission electron micrographs of the kiwifruit juice fortified with (**A**) no liposomes, (**B**) free green tea waste extract (GTWE), (**C**) empty liposomes, (**D**,**E**) GTWE-loaded single layer liposomes (SLLs) without pasteurisation, and (**E**,**F**) GTWE-loaded SLLs with pasteurisation. Magnification: 6000× for (**D**,**F**) and 43,000× for (**A**–**C**,**E**,**G**).

As can be seen in Figure 2, the soluble dietary fibres in the kiwifruit juice, which was mostly comprised of pectin (472–708 mg/100 FW) [26], appeared in the A–G micrographs. Such morphology is in line with the findings of Sorrivas et al. [27], who reported almost the same microstructure for apple fibres. The pectin structure is an open network with single or multiple strands formed as loose or bundle structures [27]. It is also notable that when free GTWCs were added to the kiwifruit juice, the microstructure was different than the juice without any catechins (Figure 2, A vs. B). The morphological data found for liposomes added to kiwifruit juice in this study agreed with those reported by Rashidinejad, Birch, Sun-Waterhouse, and Everett [3], who incorporated green tea catechin liposomes into a low-fat hard cheese. In the current study, the empty liposomes gained a slightly smaller particle size than the SLLs (Figure 2, C vs. D). Most importantly, pasteurisation affected the size of the liposomes in such a way that the liposomes in the pasteurised juice appeared bigger.

The reasons for this phenomenon were explained in Section 2.1.1, where the effect of pasteurisation on the size of liposomes in the kiwifruit juice was discussed. In brief, the presence of Ca^2+^ can result in the aggregation of liposomes because of the dehydration of the bilayers that can occur due to the induction of Ca^2+^, which in turn can be accelerated by the thermal treatment (i.e., pasteurisation). Additionally, the interactions between the fibre and phospholipid polymer chains were involved in the increase in the particle size because the SLLs might have been degraded and destroyed during the pasteurisation process, leading to the disentanglement of their phospholipids. This, in turn, resulted in the liberation of the polymer chains with the possibility of reacting with the fibre in the kiwifruit juice.

#### 2.1.4. Concentration of Catechins Determined by HPLC Analysis

**Figure 3:**
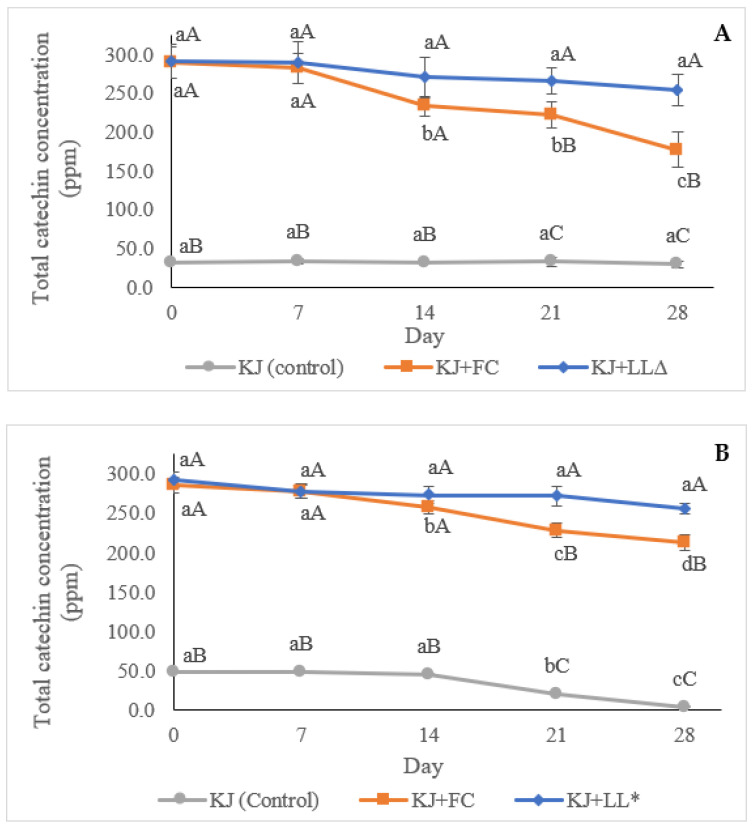
Total catechin concentration (ppm) of the control kiwifruit juice vs. unpasteurised (**A**,**B**) and pasteurised kiwifruit juice samples, analysed by high-performance liquid chromatography (HPLC). KJ: control kiwifruit juice; KJ + FC: kiwifruit juice containing free green tea waste catechins (GTWCs); KJ + LL∆: unpasteurised kiwifruit juice enriched with green tea waste extract (GTWE)-loaded single layer liposomes (SLLs); KJ + LL*: pasteurised kiwifruit juice containing GTWE-loaded SLLs. Different lowercase letters indicate significant (*p* < 0.05) differences among the same kiwifruit juice samples for different storage times. Different capital letters stand for statistically significant (*p* < 0.05) differences among different kiwifruit juice samples on the same storage day.

As shown in Figure 3, in the case of the kiwifruit juice containing SLLs, pasteurisation slightly affected the concentration of total catechins in green tea waste during the 28 days of juice storage. In contrast, pasteurisation played a crucial role in the degradation of natural and free GTWCs in the control and fortified kiwifruit juice samples, respectively. The unpasteurised kiwifruit juice maintained the same quantity of natural catechins (about 29.17 ± 4.40 to 33.46 ± 3.39 ppm) during the entire storage period, while the pasteurised kiwifruit juice showed a higher concentration of catechins (45.12 ± 10.51 to 48.83 ± 3.54 ppm) than the unpasteurised kiwifruit juice until Day 14; however, the catechin concentration drastically decreased to 19.27 ± 5.07 and 3.53 ± 1.08 on Days 21 and 28, respectively.

In terms of the kiwifruit juice containing free GTWCs, a steady decrease was observed from Day 0 to Day 28 in the case of both the pasteurised and unpasteurised samples, except for the unpasteurised kiwifruit juice on Day 28, which presented a drastic decrease for free GTWCs. These results indicate that the liposomal encapsulation could protect GTWCs during the pasteurisation process of the kiwifruit juice and until 28 days of storage, while the kiwifruit juice samples enriched with free GTWCs showed a continuously decreasing trend over time. Even though the natural GTWCs in kiwifruit juice treated with pasteurisation were mostly degraded after 14 days of storage, the unpasteurised sample could remain the same quantity until the end of the storage period.

Regarding the discrepancy of the values for the total catechins between the pasteurised and unpasteurised control kiwifruit juice samples during storage observed in this study, Tembo et al. [28], who investigated the influence of thermal processing (72 °C, 15 s) on epicatechins in the pasteurised baobab juice stored at 6 °C for 60 days, reported that the pasteurisation could enhance the concentration of epicatechins in the juice about 10% and 70% on Days 0 and 28 of storage, respectively, compared with the unpasteurised juice kept under the same condition. In the case of the current study, after Day 28, the level of epicatechins gradually decreased until Day 60. The reasons for this phenomenon originated from the impact of the pasteurisation on the loss of the polyphenol−dietary fibre bonds, and the epimerisation and the deterioration of polyphenols. The complex of polyphenol−dietary fibre could be broken by high-temperature process, leading to the release of polyphenols from the dietary fibre [29]. Accordingly, the thermal processing could result in the acceleration of catechin epimerisation from epimerised catechins (i.e., EC, ECG, EGC, and EGCG) to non-epimerised catechins (i.e., (±)-catechins (C), (−)-catechin gallate (CG), (−)-gallocatechin (GC), and (−)-gallocatechin-3-gallate (GCG)) at the C-2 position (Ananingsih et al., 2013). Moreover, the non-epimerised catechins rapidly degraded because of their poor stability [30].

The reason for the decrease in total catechins during storage in the kiwifruit juice containing free GTWE treated by pasteurisation was related to the influence of pasteurisation on the stability of phenolic compounds. In this regard, Igual et al. [31] studied the total phenols of grapefruit juice after pasteurisation (95 °C, 10 s) and stored at 4 °C for two months. The results demonstrated that the total phenols of pasteurised grapefruit juice decreased constantly (from about 70 to about 50 mg GAE/100 mL on Days 0 and 25, respectively) and remained stable until Day 60. This may agree with the decrease in the catechin content of the kiwifruit juice samples in the current study. Therefore, it can be concluded that pasteurisation could substantially decrease the total catechins of the kiwifruit juice during storage and under refrigeration conditions. Therefore, low-temperature pasteurisation or non-thermal processing may be better options for the pasteurisation of such functional foods containing green tea catechins.

In terms of the stability of the liposomes after thermal treatment, Feng et al. [32] investigated the impact of pasteurisation (65 °C, 30 min) on low-methoxyl pectin-coated liposomes containing EGCG and resveratrol in orange juice using the thiobarbituric acid reactive substance (TBARS) assay. TBARS values could measure lipid peroxidation, which was determined by the detection of malondialdehyde (MDA), so it could be implied that the higher the levels of MDA, the greater the degradation of the liposomes. The results demonstrated that pasteurisation did not considerably influence the stability of liposomes because the TBARS values of pasteurised orange juice samples, kept at 4 °C for 0, 10, and 20 days, presented a slight increase during storage (approximately 4 µM per 10 days). Moreover, the leakage percentage of the EGCG liposomes was 5.69 and 13.53% on Days 10 and 20, respectively, which may correspond with the decrease in values of the total catechin content observed during the current study (Figure 3).

#### 2.1.5. Total Antioxidant Activity (TAA)

**Figure 4:**
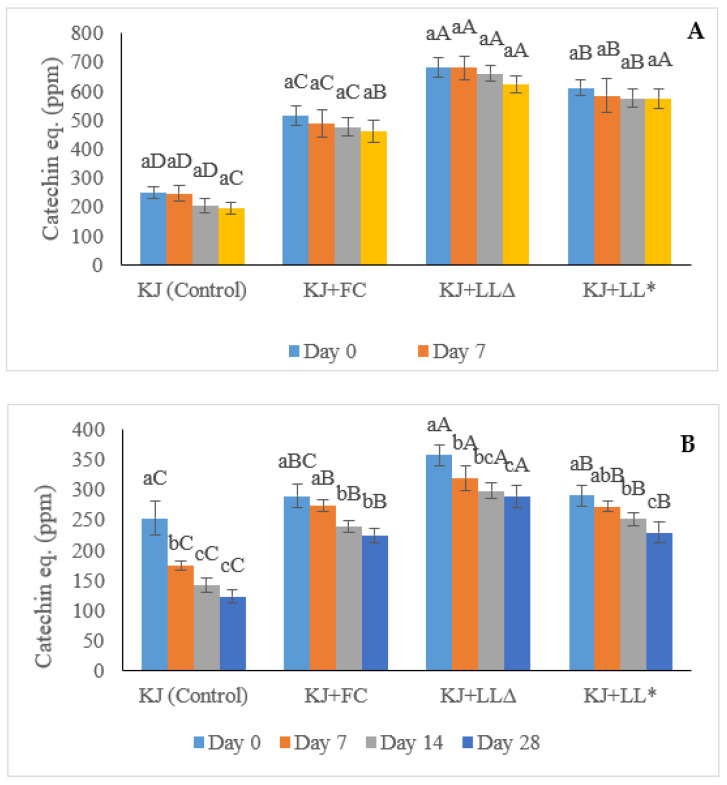
DPPH (2,2-diphenyl-1-picryl-hydrazyl) antioxidant activity (**A**) and total phenolic content (TPC) (**B**) of kiwifruit juices during 28 days of storage, expressed as catechin equivalent (ppm). KJ: control kiwifruit juice; KJ + FC: kiwifruit juice containing free green tea waste extract (GTWE); KJ + LL∆: unpasteurised kiwifruit juice enriched with GTWE-loaded single layer liposomes (SLLs); KJ + LL*: pasteurised kiwifruit juice containing GTWE-loaded SLLs. Different lowercase letters indicate significant (*p* < 0.05) differences among the same kiwifruit juice samples for different storage times; different capital letters stand for statistically significant (*p* < 0.05) differences among different kiwifruit juice samples on the same storage day.

Although all samples showed a slight reduction in the DPPH antioxidant activity during the 28 days of storage (Figure 4A), the kiwifruit juice samples supplemented with the SLLs had significantly (*p* > 0.05) higher DPPH free radical scavenging ability, ranging from 575.07 ± 33.45 to 681.54 ± 32.60 ppm than both the control kiwifruit juice and the kiwifruit juice enriched with free GTWCs (486.22 ± 37.93 to 599.53 ± 45.51 ppm). In the case of the kiwifruit juice samples fortified with the SLLs, the unpasteurised sample showed significantly (*p* < 0.05) greater DPPH values than the pasteurised sample until Day 14, while the DPPH results of both samples on Day 28 were not significantly (*p* > 0.05) different. These results are consistent with those reported by Kim et al. [33], who investigated the influence of storage temperature on the antioxidant activity of green tea catechins using the DPPH assay. In their study [33], the DPPH radical scavenging activity (%) of green tea powder kept at 4 °C was not found to be significantly (*p* > 0.05) different than the green tea powder stored at 25 and 35 °C until Day 14 (>80% and 75.42% on Days 0 and 14, respectively), but a significant decrease was observed in those samples after Day 21.

The reasons for the decrease in the TAA of the samples during storage in this study were possibly related to the epimerisation and stability of the catechins. As reported by Xu, Yeung, Chang, Huang, and Chen [30], the epimerised catechins consisting of EC, ECG, EGC, and EGCG had higher antioxidant activity than the non-epimerised catechins such as C, CG, GC, and GCG, and the epimerisation process was accelerated by heat treatment including pasteurisation. Therefore, the high temperature could be a cause of the changes in the structures of catechins from epimerised to non-epimerised forms that resulted in the reduction in antioxidant activity. Rashidinejad et al. [34] presented the influence of catechin addition on the antioxidant properties of a low-fat hard cheese. The cheese containing different concentrations of catechins (125, 250, and 500 ppm) was kept at 8 °C and examined on Days 0, 30, and 90 for the possible changes in its TAA by the DPPH method. The results of all concentrations showed an increase in TAA during the three months of storage, resulting in the release of antioxidants from cheese regardless of the change in the catechin structure. It was speculated that almost all catechins added to the cheese were stable at 8 °C during the 90 days of storage [34]. These findings correspond to the discrepancy of the TAA between the pasteurised and unpasteurised kiwifruit juice as well as the stability of GTW catechins during 28 days of storage, as presented in this study. Nevertheless, the behaviour of catechins in a beverage system such as the kiwifruit juice experimented on in this study can be different to that in a complex food matrix such as the cheese experimented on by Rashidinejad, Birch, Sun-Waterhouse, and Everett [34].

The difference between the TAA of the kiwifruit juice fortified with free GTWE and the SLLs that originated from the improvement in the stability of catechins was the result of the nanoencapsulation technology, and possibly the synergistic effect between the catechins and vitamin C (present in kiwifruit juice). Dube et al. [35] reported that the half-initial concentration degradation of free green tea catechins was faster than the encapsulated catechins in the period of 8 and 24 h. The kinetics of both catechins were also measured by k values represented as a degradation rate constant. The degradation rate of free catechin was −0.09 and −0.11%/h compared to −0.03 and −0.02%/h for the encapsulated catechin and EGCG, respectively. Toro-Uribe et al. [36] investigated the liposomal stability of procyanidins (extracted from cocoa) by measuring TAA (DPPH method). Procyanidins originate from the formation of catechin and epicatechin monomers and are usually found in cocoa, tea, and apple. The results of DPPH indicated that the encapsulated procyanidins gained about a 91.31% DPPH reduction; whereas free procyanidins only received about 44.83% DPPH reduction during 8 h of the simulated duodenum digestion. This led to the speculation that the encapsulation technique successfully delivered procyanidins to the duodenum with high TAA while over half of the free procyanidins were degraded.

Regarding the synergistic effect between catechins and vitamin C, Dube et al. [35] compared the degradation of the individual catechin (control) with the combination of catechin and vitamin C. The control and the mixture of catechin and vitamin C were prepared in phosphate buffer (pH 7.4) and the remaining catechins were observed over time. The remaining catechins in the control was about 67.5 and 19.2% at 6 and 24 h, respectively, which was substantially lower than the remaining catechins in the mixture of catechin and vitamin C (79.1 and 38.1% at 6 and 24 h, respectively). In an almost similar experiment, Saucier and Waterhouse [37] studied the synergistic effect of vitamin C and (+)-catechin. The results showed that the combination of (+)-catechin and vitamin C gained a significantly (*p* < 0.05) higher antioxidant capacity than that of either (+)-catechin or vitamin C alone, meaning that the synergistic effect of these compounds could enhance the total antioxidant capacity.

#### 2.1.6. Total Phenolic Content (TPC)

The Folin–Ciocâlteu method was used in this study for the determination of TPC, which was valuable for the confirmation of the results received by HPLC analysis and the DPPH assay. The TPC values of all kiwifruit juice samples including the control were decreased during the storage period (Figure 4B). The TPC results from the unpasteurised kiwifruit juice fortified with the SLLs possessed the highest values with statistical significance at every storage point (i.e., Days 0, 7, 14, and 28), ranging from about 289.4 ± 17.78 to about 357.83 ± 17.41 ppm of catechins equivalent, followed by the pasteurised kiwifruit juice fortified with the SLLs. These results agree with the findings of Zhao and Shah [38], who presented the effect of storage time on the TPC concentration of fermented soymilk enriched with GTE. The TPC concentration of fermented soymilk containing GTE continuously decreased from 4.31 to 3.71%, meaning that the tea phenolics were stable for 28 days of storage at 4 °C.

The decrease in TPC in all kiwifruit juice samples during storage was associated with the decrease in the compounds that may not be antioxidant but still interact with the Folin–Ciocâlteu reagent as well as the initial degradation of compounds such as vitamin C that contribute to the antioxidant activity of kiwifruit juice. Some compounds such as amino acids, reducing sugars, and vitamin C can interact with the Folin–Ciocâlteu reagent and as they degrade over the storage period, the TPC values measured for the kiwifruit samples also decrease. It has been reported that about 70–80% of vitamin C from orange could degrade within 8 h after the squeeze, even when it was kept in the refrigerated condition (8 °C) [39]. Furthermore, the previous research showed that the proper storage temperature and time (4 °C for 28 days, respectively) could not have a negative effect on the TPC results in green tea polyphenols [40,41]. For these reasons, in this experiment, the drastic reduction in TPC on Day 7 possibly resulted from the degradation of other compounds in kiwifruit juice (e.g., reducing sugars and vitamin C), while the TPC reduction for the rest of the storage period mostly resulted from the decrease in GTW catechins.

### 2.2. The Behaviour of the Double-Layer Liposomes (DLLs) in Green Kiwifruit Juice

#### 2.2.1. Particle Size and Zeta Potential Changes

**Figure 5:**
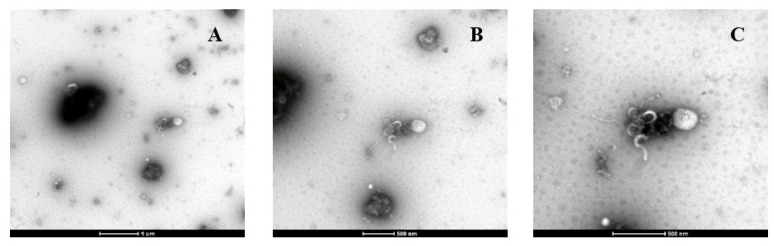
Transmission electron micrographs of the kiwifruit juice containing the double-layer liposomes at different magnification: (**A**) 16,500×, (**B**) 26,500×, and (**C**) 43,000×.

As shown in Table 1, the size of the particles in the kiwifruit juice containing the DLLs (produced from soy lecithin and chitosan) was significantly higher than that of the control juice (approximately 2.90 µm) at any point of the storage period, especially on Day 28 (around 22.62 µm). This could be attributed to the presence of calcium ions (Ca^2+^), pectin methylesterase (PME) activity, and the electrostatic attraction between the DLLs and pectin polysaccharides. Wicker et al. [42] studied the effect of PME and calcium ions on the clarification of orange juice, and reported that the cloud stability of orange juice stored at 4 °C was eight days for the juice containing PME and calcium ions and 13 days for the control juice. The addition of PME and calcium ions increased the particle size of the orange juice up to 30 µm (D[4, 3]-Volume weighted mean) after eight days [42]. Therefore, in the case of the present study, the presence of PME and calcium ions (approximately 34 mg/100 g FW) in kiwifruit juice might be one of the responsible factors for increasing the size of the particles in the kiwifruit juice fortified with the DLLs. The DLLs contain positive charges while pectin in kiwifruit juice contains negative charges; thus, they can interact with each other via electrostatic force [27,43]. This reaction can be considered as another cause of the increase in particle size observed since Day 0, which can be clearly explained by the TEM micrographs presented in Section 2.2.2 (Figure 5).

The zeta potential of the kiwifruit juice containing the DLLs was almost as same as the control juice throughout the storage period (Table 1). This might be because the concentration of the DLLs added in the kiwifruit juice was not high enough to influence the surface charge of the particles in the juice. In other words, it is possible that the negative charges of the kiwifruit juice particles dominated the positive charges of chitosan. Therefore, there was no significant difference between the zeta potential of the control kiwifruit juice and the kiwifruit juice containing the DLLs. These results are partially in line with the findings of Rashidinejad, Birch, and Everett [12], who measured the zeta potential of the C, EGCG, and GTE added to the milk fat globule dispersions at different concentrations (125, 250, 500, and 1000 ppm). Based on their study [12], in the case of the low concentration of catechins, the zeta potential of the milk fat dispersion did not significantly change (*p* > 0.05). For example, the zeta potential of milk fat dispersion fortified with catechins in the concentration of 125, 250, and 500 ppm ranged from about −12.70 to −12.67 mV, which was not significantly different (*p* > 0.05) from the pure milk fat (control dispersion; −12.67 mV), but the milk fat containing 1000 ppm catechins gained a surface charge of −13.34 mV, which was significantly (*p* < 0.05) higher than the control. Based on this evidence, the concentration of the DLLs in the current study may not have been high enough to change the zeta potential of the kiwifruit juice.

#### 2.2.2. Morphology and Microstructure 

According to Figure 5, the DLLs were attached to the fibre in kiwifruit juice, possibly due to the electrostatic attractions between the positively charged DLLs and negatively charged pectin. The morphology of the DLLs and fibre seen in the current study was close to that reported by Mady et al. [44] as well as those presented by Sorrivas, Genovese, and Lozano [27], who reported the morphology of chitosan-coated liposomes and apple fibre, respectively. The interaction between the DLLs and kiwifruit fibre could form the DLL–pectin complex, which could have possibly been the cause of the increase in particle size, as reported in Section 2.2.1. This complex may lead to aggregation, which in turn, results in the instability of DLLs [45].

#### 2.2.3. Total Phenolic Content (TPC) and Total Antioxidant Activity (TAA)

As can be seen from the results presented in Table 1, the kiwifruit juice containing the DLLs showed significantly higher TPC values than both the control kiwifruit juice and the kiwifruit juice fortified with free GTWCs (*p* < 0.05) on Day 0. However, from Day 7 until the end of the storage period (i.e., Day 28), the TPC values of the kiwifruit juice containing the DLLs were not significantly different (*p* > 0.05) than the kiwifruit juice fortified with free GTWCs, although still higher than the TPC value of the control juice.

In terms of the TAA analysed by the DPPH assay like the TPC values, on Day 0, the kiwifruit juice containing the DLLs presented significantly (*p* < 0.05) higher values than both the control and the kiwifruit juice fortified with free GTWCs. However, there was no significant difference between the kiwifruit juice containing the DLLs and the kiwifruit juice fortified with free GTWCs after seven days of storage. These values were lower than those reported in Section 2.1.4 and Section 2.1.5 because of the difference in the type of liposomes that showed different stability after their incorporation into the kiwifruit juice. It could be presumed that the SLLs, which were only made of soy lecithin, had more potential to protect GTW catechins in kiwifruit juice than the DLLs made of soy lecithin and chitosan. Possibly, the repulsion force between soy lecithin liposomes and fibre in kiwifruit juice could protect the aggregation and precipitation processes, leading to better stability of the SLLs. On the other hand, the interactions between chitosan in the case of the DLLs and fibre in kiwifruit juice resulted in the instability of such particles. Therefore, it can be said that in the case of this study, in terms of the protection of GTW catechins in kiwifruit juice, the SLLs were more efficient than the DLLs. Nonetheless, this cannot be generalised, meaning that the DLLs could be more efficient for the incorporation of some other bioactives and/or into some other food formulations. In this study, only one hypothesis was tested, which was based on the information available in the literature. Based on the findings available in the literature, a three-layer liposomal system might be effective in the protection of GTW catechins in kiwifruit juice. Since the surface charge of the triple-layer liposomes is negative, it can generate a repulsion force between the liposomes and kiwifruit fibre. It may be predicted that the physical stability of triple-layer liposomes is higher than that of secondary liposomes; nevertheless, it is uncertain whether triple-layer liposomes can protect green tea catechins more efficiently than DLLs in a food system such as green kiwifruit juice, which is currently under investigation in our laboratories.

## 3. Materials and Methods

### 3.1. Green Tea Waste, Kiwifruit, Reagents, and Chemicals

Dried green tea waste (Guiding Niaowang) was obtained from Guizhou Eight Grams Tea and Agricultural Development Ltd. (Qiannan, China). Green kiwifruit was purchased from a local supermarket (PAK’nSAVE, Palmerston North, New Zealand) and stored at 4 °C until used. The high purity (HPLC grade) catechin standards including catechin (C), epicatechin (EC), epicatechin gallate (ECG), epigallocatechin (EGC), and epigallocatechin gallate (EGCG) were purchased from Sigma-Aldrich, Inc. (Darmstadt, Germany). Low viscosity light liquid soy lecithin (Beakin^®^ LV1 Lecithin) was purchased from Archer Daniels Midland Co. (Chicago, IL, USA). 2,2-Diphenyl-1-picryl-hydrazyl (DPPH) reagent was procured from Sigma-Aldrich Co. Inc. (Darmstadt, Germany). Folin–Ciocâlteu reagent was purchased from Merck Co. Inc. (Rahway, NJ, USA). All other chemicals and reagents used in this study were of analytical grade.

### 3.2. Preparation of Liposomes

The preparation of single-layer liposomes was adapted from the method developed by Rashidinejad, Birch, Sun-Waterhouse, and Everett [3] with slight modifications. Soy lecithin (1% *w*/*v*) was dispersed in the solution of green tea waste extract (GTWE) and acetate buffer and stirred with a magnetic stirrer (300 rpm) for 30 min. The dispersion was then mixed using a high-speed mixer (D500 series, Labserv, Langenselbold, Germany) at 24,000 rpm (5 × 1 min bursts). The double-layer liposomes were prepared following the method of Laye et al. [46] with some modifications. The preparation of chitosan solution started by dissolving 0.1% (*w*/*v*) chitosan in the acetate buffer (pH 3.8), followed by overnight stirring (300 rpm). To create chitosan-coated liposomes, the single-layer liposomes (0.5% *w*/*v*) were added to the chitosan solution and stirred (700 rpm) for 2 min at 20 °C. The prepared liposomes were stored at 4 °C for 24 h before further investigation and use.

Two controls and one blank were prepared for comparison. The first control was the GTWE solution without lecithin and the second control contained 1% *w*/*v* of soy lecithin dispersed in the same acetate buffer without the addition of GTWE. The blank consisted of only acetate buffer (i.e., no GTWE or soy lecithin). The controls and blank were processed in the same way as the treated samples (i.e., same magnetic stirring and homogenisation processes).

#### 3.2.1. Encapsulation Efficiency

Liposomes containing SLLs were assessed for the encapsulation efficiency of green tea catechins, based on the method adapted from that of Rashidinejad, Birch, Sun-Waterhouse, and Everett [3]. The prepared liposomes were filtered by Sephadex G100 gel filtration. When the prepared liposomes were passed through the gel filtration, non-encapsulated catechins were entrapped in the gel, while the encapsulated catechins passed through the column. The encapsulation efficiency was calculated using the equation below:

(1) $$F_{i\;}\left(\%\right)=\frac{C_{\mathrm{total}}-C_{\mathrm{out}}}{C_{\mathrm{total}}}\times 100$$

where F_i_ = the encapsulation efficiency depending on the concentration of catechins in the liposome structure; C_total_ = the total concentration of both encapsulated and non-encapsulated catechins; and C_out_ = the concentration of non-encapsulated catechins.

#### 3.2.2. Loading Capacity

The loading capacity, which represents the maximum mass of catechins that can be encapsulated in liposomes [20], was calculated using the equation below:

(2) $$\mathrm{Loading}\;\mathrm{capacity}\;\left(\%\right)=\frac{C_{\mathrm{total}}-C_{\mathrm{out}}}{\mathrm{weight}\;\mathrm{of}\;\mathrm{capsules}}$$

where C_total_ = the total concentration of both encapsulated and non-encapsulated catechins, and C_out_ = the concentration of non-encapsulated catechins.

### 3.3. Manufacture of Green Kiwifruit Juice and the Incorporation of the Encapsulated Catechins

The preparation of green kiwifruit juice was adapted from the method reported by Guo et al. [47] with slight modifications. Green kiwifruit was peeled, and the pulp was squeezed by a juicer (Breville Group Ltd., Juice Fountain JE95, Sydney, Australia, 1000 W). The juice was centrifuged at 4000 rpm for 10 min (22 °C) to separate big particles. The supernatant was collected and passed through a 100 µm filter to remove the remaining pulp. Afterward, the encapsulated GTWE (in addition to the control and blank samples) was added to the manufactured kiwifruit juice at the concentration of 280 ppm. The enriched kiwifruit juice was divided into two portions. The first portion underwent the pasteurisation process (80 °C for 30 s) [48], while the second portion was not pasteurised. Pasteurised and unpasteurised samples were then divided into four portions in plastic containers covered with aluminium foil. These samples were used for the assessment of the behaviour of the encapsulated catechins during a certain period of storage (0, 7, 14, and 28 days) at 4 °C.

### 3.4. The Behaviour of the Encapsulated GTWE in Green Kiwifruit Juice and the Effect on Its Properties

#### 3.4.1. Particle Size and Zeta Potential Measurements 

The particle size of the manufactured liposomes was determined using a Zetasizer (Malvern Instruments Ltd., nano ZS, Malvern, UK). In the case of the kiwifruit juice fortified with the liposomes, the particle size was measured by a Mastersizer (Malvern Instruments Ltd., Hydro 2000 MU, Malvern, UK). The zeta potential of both liposomes and the kiwifruit juice containing liposomes was studied using a Zetasizer (Malvern Instruments Ltd., nano ZS, Malvern, UK). The samples that were analysed by the Zetasizer were diluted (1:16) in 0.25 M acetate buffer (pH 3.8), while in the case of the Mastersizer measurements, no dilution was required [3].

#### 3.4.2. Morphology Characterisation

Transmission electron microscopy (TEM) was used to examine the morphology of the manufactured liposomes. The sample preparation using the negative staining technique was slightly modified from the method developed by Ruozi et al. [49]. Liposome samples were diluted 20 times before placing a small droplet (~80 µL) on the parafilm. A 200-mesh formvar copper grid (Agar Scientific Ltd., Essex, UK) was placed into the liposome droplet for 4 min. To stain the grid, a drop of 2% uranyl acetate was placed on the parafilm and the grid (coated with liposomes) was incubated in the solution for 4 min. The stained copper grid needed approximately 2 min for drying at room temperature. Finally, the microstructure of the liposomes was seen under TEM (FEI Tecnai G2 Spirit BioTWIN, Brno-Černovice, Czech Republic) at 100 kV. A Veleta CCD camera (Olympus Soft Imaging Solutions, Munster, Germany) was used to capture the images.

#### 3.4.3. HPLC Analysis

Catechins were analysed based on the method reported by Wang et al. [50] with slight modifications. An Agilent 1200 series high-performance liquid chromatography (HPLC) apparatus equipped with a C18 reversed-phase Synergi 4 µm (150 × 4.6 mm) was used. The mobile phase was composed of Eluent A (containing 0.1% orthophosphoric acid in water *v*/*v*) and Eluent B (containing 0.1% orthophosphoric acid in methanol *v*/*v*). The gradient was set as 0–5 min, 20% B; 5–7 min, linear gradient from 20 to 24% B; 7–10 min, 24% B; 10–20 min, linear gradient from 24 to 40% B; 20–25 min, linear gradient from 40 to 50% B. Post-run time was 1 min and the flow rate of the eluents was controlled at 0.8 mL/min, with an injection volume of 5 µL. The column temperature was kept at 30 °C. In terms of detection, a diode array detector was used at the wavelengths of 210 and 280 nm. The identification of catechins was based on the retention times of their peaks and the UV–Vis spectra compared with the calibration curve made from the five standards (i.e., C, EC, ECG, EGC, and EGCG) at the concentrations of 2–50 ppm.

#### 3.4.4. Total Phenolic Content

The total phenolic content (TPC) was investigated by applying the method developed by Du et al. [51] with some modifications. The Folin–Ciocâlteu stock solution was prepared by mixing the Folin–Ciocâlteu reagent with distilled water in a ratio of 1:1. Liposome samples were diluted at the ratio of 1:10 (*v*/*v*) with methanol, while kiwifruit juice samples containing the encapsulated GTWE were ready for analysis without any dilution. A total of 0.5 mL of the Folin–Ciocâlteu reagent was mixed with 7.9 mL of distilled water and 0.1 mL of the prepared liposomes or kiwifruit juice samples. After 1 min, 1.5 mL of sodium carbonate (1:5 *w*/*v*) was added before the sample was vortexed and kept in the dark (room temperature) for two hours. The absorbance was measured by a spectrophotometer (GENESYS 10 Series, UV–Vis, WI, USA) at 765 nm and TPC values were calculated based on the calibration curve made using (+)-catechin as the standard (0.20–25 ppm).

#### 3.4.5. Total Antioxidant Activity (TAA) Determination

The DPPH (2,2-diphenyl-1-picryl-hydrazyl) free radical method was applied according to the method reported by Kara and Erçelebi [52] with some modifications. The DPPH stock solution was prepared by dissolving DPPH 1.2 mg in methanol (50 mL). Liposome samples were diluted at the ratio of 1:10 (*v*/*v*) with methanol, while kiwifruit juice samples were ready for analysis without any dilution. A total of 0.1 mL of the prepared samples was mixed with 3.9 mL of DPPH solution. The mixtures were kept in the dark at room temperature for 30 min. After that, the absorbance was determined by a spectrophotometer (GENESYS 10 Series, UV-vis, WI, USA) at 515 nm. The %DPPH reduction was calculated using the formula below:

(3) $$\%\mathrm{DPPH}\;\mathrm{reduction}=\left(\frac{A_{c}-A_{s}}{A_{c}}\right)\times 100$$

where A_C_ = the absorbance of the control (*t* = 0 min); A_S_ = the absorbance of the tested sample at the end of the reaction (*t* = 30 min).

All measurements were carried out at room temperature (25 °C). The %DPPH of the samples was calculated based on the calibration curve made using (+)-catechin as the standard (0.20–25 ppm) [53].

### 3.5. Statistical Analysis

The reported data are the means of at least three measurements. All measurements were performed in triplicate. Minitab (Version 17.3.1) Statistical Software (Minitab Inc., State College, PA, USA) was used to carry out the corresponding statistical analysis. The data were subjected to the analysis of variance (ANOVA) for the mean comparison for any significant differences (*p* < 0.05). All graphical presentations were generated by Microsoft Excel 2016 (Microsoft Corporation, Redmond, WA, USA).

## 4. Conclusions

The findings of this study revealed the stability of both SLLs and DLLs containing GTWCs during the storage period as well as the feasibility of their incorporation into a functional fresh green kiwifruit juice. The SLLs possessed high negative surface charge values together with a small particle size, demonstrating their strong physical stability up to the end of the storage period (28 days). On the other hand, the DLLs showed a larger particle size and high positive zeta potential values. The SLLs had a higher potential to maintain the stable stage of liposomes than the DLLs in the kiwifruit juice because the negative surface charges of the SLLs could repel kiwifruit fibre molecules to prevent aggregation and degradation. Additionally, in kiwifruit juice, the TPC and TAA of the DLLs were lower when compared with the SLLs. This was possibly due to the leakage of the DLLs from the sedimented liposomes (caused by the interactions between chitosan and fibre) and their consequent degradation. Further research in our laboratories is focused on the development of triple-layer liposomes (TLLs) containing GTWCs and their incorporation into kiwifruit juice (and similar delivery vehicles). Such vesicles may provide better stability than SLLs and DLLs due to better protection of the aggregation and precipitation processes. In addition, the behaviour of such a developed ingredient in various functional food products and the bioaccessibility/bioavailability of catechins after food consumption are yet to be investigated.

## Figures and Tables

**Table 1:** The particle size (D [4, 3]-Volume weighted mean (µm)), zeta potential (mV), total phenolic content (ppm), and total antioxidant activity (ppm) of kiwifruit juice containing the double-layer liposomes (DLLs) during the 28 days of storage at 4 °C.

Analytical Methods/Storage Period	Day 0	Day 7	Day 14	Day 28
*Particle size* *(D [4, 3]-Volume weighted mean (µm))*				
Kiwifruit juice (control)	2.85 ± 0.04 ^aB^ *	2.90 ± 0.05 ^aB^	2.88 ± 0.04 ^aB^	2.91 ± 0.02 ^aB^
Kiwifruit juice + DLLs	3.23 ± 0.07 ^bA^	3.34 ± 0.08 ^bA^	3.27 ± 0.11 ^bA^	22.62 ± 1.71 ^aA^
*Zeta potential (mV)*				
Kiwifruit juice (control)	−8.80 ± 0.46 ^aA^	−8.30 ± 0.55 ^aA^	−8.45 ± 0.46 ^aA^	−9.06 ± 0.79 ^aA^
Kiwifruit juice + DLLs	−8.98 ± 0.46 ^aA^	−8.77 ± 0.49 ^aA^	−8.11 ± 0.52 ^aA^	−8.88 ± 0.66 ^aA^
*Total phenolic content* *(catechin equivalent (ppm))*				
Kiwifruit juice (control)	253.30 ± 27.40 ^aB^	174.09 ± 7.83 ^bB^	141.16 ± 11.94 ^cB^	122.29 ± 10.90 ^cB^
Kiwifruit juice + free GTW catechins	290.37 ± 19.11 ^aB^	273.59 ± 9.28 ^aA^	238.90 ± 10.06 ^bA^	224.24 ± 11.83 ^bA^
Kiwifruit juice + DLLs	359.86 ± 30.65 ^aA^	263.99 ± 9.39 ^bA^	226.44 ± 8.07 ^cA^	226.12 ± 11.36 ^cA^
*Total antioxidant activity* *(Catechin equivalent (ppm))*				
Kiwifruit juice (control)	248.64 ± 20.28 ^aC^	245.90 ± 26.90 ^aB^	205.07 ± 23.46 ^aB^	195.30 ± 18.85 ^aB^
Kiwifruit juice + free GTW catechins	514.90 ± 34.60 ^aB^	487.30 ± 46.70 ^aA^	476.20 ± 30.80 ^aA^	461.50 ± 37.90 ^aA^
Kiwifruit juice + DLLs	555.08 ± 26.58 ^aA^	488.21 ± 11.56 ^bA^	480.24 ± 10.27 ^bA^	472.67 ± 18.35 ^bA^

* Different lowercase superscripts indicate significant (*p <* 0.05) differences among the same kiwifruit juice samples for different storage times. Different uppercase superscripts stand for statistically significant (*p <* 0.05) differences among different kiwifruit juice samples at the same storage time.

## Data Availability

Not applicable.
